# Establishing the common ground in European psychotraumatology

**DOI:** 10.3402/ejpt.v6.28213

**Published:** 2015-05-26

**Authors:** Vedat Şar

**Affiliations:** 1 Koç University School of Medicine (KUSOM), Istanbul, Turkey. Email: vedats@amerikanhastanesi.org; 2 European Society for Traumatic Stress Studies (ESTSS), 2013–2015 Diemen, The Netherlands; 3 International Society for the Study of Trauma and Dissociation (ISSTD), 2008–2009 McLean, Washington DC, USA

The chief ethical rule is the following: thou shalt not have antifragility at the expense of the fragility of others. (Taleb, 2012)

Europe is nicely complex; that is, rich and full of diversity. Lessons learned from the painful past are immense (Betancourt, 2015) together with a healthy anxiety about the future. One may perceive Europe as the most prosperous, peaceful, and safest part of the world. Yet this rich heritage is also a reflection of a hidden truth: trauma is not only endemic in Europe, but it also lives in the soul of Europeans (Orner, 2013).

Two decades after its foundation, the premier organization of European psychotraumatologists, the European Society for Traumatic Stress Studies (ESTSS) is completing its protracted structural transformation and takes an important step to fulfill its key vision, namely, solidify international collaboration among trauma professionals throughout the whole continent. Synchronization of knowledge and experience among all participants is necessary to achieve these ends, and may itself be a creative process. At the end of this transition from a society built around voluntary individual membership to one of an umbrella organization for regional (mostly national) trauma societies, ESTSS will benefit from tremendous organizational efficiencies as a transnational venue to nurture the common ground in European psychotraumatology. To advance this, ESTSS spreads its knowledge through biannual conferences and the *European Journal of Psychotraumatology* (EJPT).

## Science in transition

The most robust change in the world of contemporary science seems to be in availability of knowledge. “Open access” journals are increasingly becoming the standard of scientific publishing. Once a domain of youngsters, experiences collected via development of “social” media pave the way to user-friendly academic portals which resemble the former in their designs. The world has turned into a “marketplace” for scientific products; a fantastic one in which goods are not paid for by customers but by producers. In this “intellectual commons,” and not withstanding continued debates and restrictions around intellectual property, knowledge is shared freely. What is the economy behind this inverted relationship? Apparently, knowledge has the tendency to grow if shared. One hopes that this endeavor diminishes the distance between knowledge and truth, and regulates the “market” in such a way that the most valuable scientific output distinguishes itself out of an almost limitless supply.

The official journal of ESTSS, the *EJPT* is a fine example of this philosophy: it is open access (Olff, 2013). Comprehensive as well as integrative in content, *EJPT* indeed represents the European flavor in the field of psychotraumatology (Olff, 2012). The achievements of *EJPT*, in December 2015 celebrating its fifth anniversary, are spectacular. It has been recognized as a high-quality scientific journal by all major scientific indexes since its inception and runs toward a noteworthy impact factor (Olff, 2014).

One wonders how the increased accessibility of science will change the world. Can it be the vehicle which might carry a “world in crisis” to a better future? Clinicians are capable of and responsible for healing individuals, but not the society as a whole. Nevertheless, society seems to be the incubator of much traumatic stress (Sar, Krüger, Martinez-Taboas, Middleton, & Dorahy, 2013). One wonders whether clinical experiences and insights derived from research on the experiences of individuals might provide leverage for change on a societal scale. In order for science to have such a societal impact (as represented, for example, by the movement of “participatory action research”), the structures and operating frameworks of scientific and professional organizations would seem to be crucial (Glassman, Erdem, & Bartholomew, 2013).

## Rise and persistence of psychotraumatology

Throughout history, pitfalls in this endeavor have been the rule rather than the exception. Jacques Lacan (1966) warned against the master's discourse which can seamlessly infiltrate the academy to turn it into a subtle medium of social repression: “Over centuries, knowledge has been pursued as a defense against truth.” Neglected by mainstream psychiatry and psychology throughout most of the past century (ironically, a period beset by every kind of trauma including the two world wars), psychotraumatology is currently experiencing a renaissance, both in Europe and globally (Schnyder, 2013). The marginalization of psychotraumatology and the subsequent “memory wars” in North America had led to concerns about the future of the field. Fortunately, history does not repeat itself when knowledge is integrated into consciousness. Despite drawbacks, psychotraumatology flourished as a science and practice. Nevertheless, this has been the result of tremendous efforts and sacrifices, and should not be mistaken as the natural outcome of a comfortable flow of events.

If the year 1980 is regarded as an anchor point (a somewhat arbitrary yet also meaningful choice in that certain chronological parallels can be drawn with respect to the professional development of the practitioners of a particular generation), several grotesque changes become apparent in shaping the modern “Zeitgeist” of psychotraumatology: The first is that, 1980 marks the start of the “globalism” which increasingly challenged national “boundaries,” at economic and cultural levels. Free market values and consumerism began to prevail against economic protectionism, and an influx of North American culture started to shatter familiar cultural values. It was also during the 1980s that the European Union welcomed Greece, Spain, and Portugal as member states, which constituted a first step of departure from its predominantly northwestern European identity. The end of “Cold War” led to the reunion of the greater Europe, but not without complications. Increased accessibility of the Internet to a much wider user base has provided tremendous opportunities enabling knowledge to seamlessly cross borders. Last but not least, 1980 was the year of publication of the North America-based highly influential document of psychiatry, namely, the DSM-III (American Psychiatric Association, 1980), a development which changed the psychiatric profession irreversibly.

The DSM-III reinstated phenomenology as the main method of describing clinical psychopathology (Jaspers, 1913) as the “common ground” in psychiatry. At the same time, it officially recognized three diagnostic categories which, in their own way, are related to psychological trauma: Posttraumatic Stress Disorder (PTSD), Dissociative Identity Disorder (aka multiple personality), and Borderline Personality Disorder. Although the first has been associated explicitly with acute traumatic stress, the latter two have been increasingly recognized to be consequences of chronic developmental trauma (Sar, 2011). Growing insight into the process-like nature of any posttraumatic response has alerted clinicians and researchers to a particular aspect of traumatic experience: namely, that there is nothing acute. (Meaning that, any traumatic situation is embedded in a larger and interdependent context of things, which requires a longitudinal perspective for holistic understanding.) This notion has since become subsumed under the rubric “complex trauma” (Courtois, 2004; Herman, 1992; Van der Kolk, 1996). In DSM-5 (American Psychiatric Association, 2013), and explicitly in the proposed ICD-11, “complex” aspects of posttraumatic stress reactions have now been included (Cloitre, Garvert, Brewin, Bryant, & Maercker, 2013; Cloitre, Garvert, Weiss, Carlson, & Bryant, 2014; Olff et al., 2015).

## Learning from tragedies: disasters and accidents

Yet trauma is a dialectical phenomenon (Fischer & Riedesser, 1999); that is, the opposite of the statement is also true. Namely, not every trauma is “complex” (e.g., an accident), at least at the beginning. The phrase is well known to every clinician: “Everything in my life has changed after this particular event.” Such events enter into one's life “all of a sudden” and unexpectedly. Yet, they constitute a turning point (Sar & Ozturk, 2005); for example, disasters and accidents have huge repercussions in individuals’ and societies’ lives for a prolonged period (Arnberg, Hultman, Michel, & Lundin, 2013).

Despite tremendous technological advancements, our tools of observation and precautionary feedback mechanisms continue to be imperfect. This makes us vulnerable to tragedies when least expected. Human failure to predict and prepare can often be the primary culprit for the destructive effects of natural disasters such as earthquakes and tsunamis or accidents such as road traffic collisions and plane crashes (Grimm, Hulse, Preiss, & Schmidt, 2012). In fact, a failure to predict often accompanies and aggravates the traumatic experience at communal and individual levels. The mass traumas created by large-scale disasters (e.g., the recent earthquake in Nepal) may have such a profound societal (as well as individual) effect that mental health professionals and governmental entities start to pronounce “trauma” in countries in which “trauma” had previously remained a marginal subject of study. The raising of awareness is usually coupled with transmission of knowledge between international and local clinicians and scientists (Reifels et al., 2013).

## Violence: more pervasive than ever?

As opposed to accidents and disasters, there is also man-made or interpersonal violence (see also Olff & Wall, 2014). Different in form than the all-out wars of the past century, violence has today taken a subtle form by penetrating daily life and constantly feeding the same subliminal message: You are never and nowhere safe. Reality has replaced the broadcasting of fictional “Star Wars”-type combats, as the latter previously replaced the “classical” theater of war. Exposure of the graphic violence on the Internet led a young adult in his twenties to believe: “I am not the same person anymore” (statement from personal conversation). Both organized and individual violence succeed in comprehensively assaulting human dignity even with respect to isolated incidents (Hicks, 2011). These phenomena routinely prevail also in and around Europe, leading to victimization of individuals and whole communities (and where refugees and itinerant workers are frequent casualties). The incidents of individual violence against civilians and children occurring from time to time in prosperous Western Europe and North America also leave unanswered questions (Thoresen, Aakvaag, Wentzel-Larsen, Dyb, & Hjemdal, 2012).

Many conflicts, wars, and violent or terrorist acts are symptomatic of a broader problem; that is, the inculcation of a resort to violence to achieve certain ends. Nevertheless, the overall effects of destruction caused by a violent act are often asymmetrical, frequently exceeding the subjective motivation, purpose, and intent of the perpetrator's act. This can be called the “betrayal of terror” (Solinski, 2014). Lloyd deMause (2002) considers war and institutional violence as a societal re-enactment of widespread traumatizing childrearing practices. He underlines the unspoken conflict and competition between different childrearing practices (“psycho-classes”) as one of the underlying factors prominent even in civil wars (Nandi, Crombach, Bambonye, Elbert, & Weierstall, 2015).

Nevertheless, where trauma prevails, dissociation lurks (Sar, Middleton, & Dorahy, 2013). Hence, both individual and societal awareness about the vicious cycle of trauma is indispensable for overcoming individual and organized violence (Levine, 1997). Breaching societal denial and silence, particularly in relation to developmental trauma (Middleton et al., 2014a), including institutional abuse (Lueger-Schuster et al., 2014; Middleton et al., 2014b), is important in nurturing awareness of this cycle. Representing the rather modest level of global consensus among clinicians and researchers prevailing in the last quarter of the past century, the limited description of chronic complex dissociative disorders in ICD-10 (World Health Organization, 1992) requires a thorough update to attune to and align with DSM-5 in this regard. Last but not least, this line of thought leads one to pondering on the ultimate goal of obtaining clinical knowledge: the ways and means of healing.

## Treatment: coupling theory with practice

Treatment of trauma-related conditions suffers from the general problems of psychotherapy: diversity of theories, models, and techniques and, not rarely, a muddle between them which leads to “confusion of therapies.” In fact, “shopping” among different approaches is often a source of anxiety for clinicians when working with their clients, and a problematic phenomenon for the medical tradition, which needs to be grounded on “authoritative” expertise and up-to-date knowledge implemented in an ethical manner.

Evidence-based practice has emerged as a motto today, which, due to the natural limits in our capacity to perceive, measure, and define phenomena, can be helpful only to a limited degree. Hence, a contrasting approach, “practice-based evidence” emerged (Duncan, Miller, Wampold, & Hubble, 2010). Given the creative nature and needs of the human spirit, this endeavor of “scientific revolutions” will and should never end (Kuhn, 1962). Historically, victims of trauma (as a natural consequence of their often uncomfortable predicament as the “oppressed”) have tended to be an agent for progress. In the sacred pursuit of healing trauma victims, it was most often clinicians dealing with trauma who thereby challenged prevailing theories and comfortable conceptions about the human psyche.

Just a historical note about this: A pupil in Salpetriere once dared the famous physician of “hysteria” (formerly the label for today's trauma-related dissociative disorders): “Dr. Charcot, what you say does not fit the theory.” The response (one of Sigmund Freud's favorite quotes) was: “Theory is good but it doesn't prevent things from existing” (Newman, 1993, p. 125). The mutual relationship between theory and practice is an exciting one. Practice is ineffective without an accompanying theory, which does not necessarily need to be perfect and can still function despite the “exceptions.” In fact, this framework is the *sine qua non* for achieving the sense of mastery both by the client and the clinician in a treatment setting. Moreover, it constitutes a necessary “common ground” for both parties and the inherently creative process the treatment entails.

The rule of thumb is that clinicians should learn how to establish mutuality between theory and practice in each particular situation, and how to make their clients and their feedback an active element of this process (Kluft, 2003). “Where I touch there I am touched as well” (Kuechenhoff, 2007). In the final analysis, what heals the human spirit is being recognized in its uniqueness as a subject rather than being considered a common object (of any theory or method).

## Conclusions

A recent roadmap for mental health research in Europe created by a group of 60 invited experts identified 20 priorities (Forsman et al., 2015). These items represent three overarching goals which mirror societal challenges: 1) to identify causes, risk, and protective factors for mental health across the life span; 2) to advance the implementation of effective public mental health interventions; and 3) to reduce disparities in mental health. Although not explicitly mentioned in this report, all items of the list are strongly influenced by traumatic stress. More than 50 published papers generated by the prospective Adverse Childhood Experiences study constitute an example demonstrating these strong links not only with mental but also bodily health (Anda et al., 2009).

Prevention, treatment, and research seem to constitute three pillars which should be essential in establishing the common ground in European psychotraumatology. Advocacy, clinical training, and sharing the methodologies embedded within each of them are likewise critical. Scientific evidence already proves that psychotraumatology research should receive funding by governmental sources as an area of research priority in terms of its implications for public health. ESTSS aims to facilitate collaboration between clinicians and researchers as well as to represent the field in the face of governmental entities (EU Treatises, 1997). These tasks cannot be carried out by national organizations of European countries in isolation and constitute the reasons why ESTSS is necessary and deserves full support by its member organizations. With two decades of experiences behind it, ESTSS is well placed to address these urgent contemporary challenges.

*Vedat Şar* Professor of Psychiatry Koç University School of Medicine (KUSOM), Istanbul, Turkey Email: vedats@amerikanhastanesi.orgPresident European Society for Traumatic Stress Studies (ESTSS), 2013–2015 Diemen, The NetherlandsFormer President International Society for the Study of Trauma and Dissociation (ISSTD), 2008–2009 McLean, Washington DC, USA

## References

[CIT0001] American Psychiatric Association (1980). Diagnostic and statistical manual of mental disorders.

[CIT0002] American Psychiatric Association (2013). Diagnostic and statistical manual of mental disorders.

[CIT0003] Anda R. F, Dong M. X, Brown D. W, Felitti V. J, Giles W. H, Perry G. S (2009). The relationship of adverse childhood experiences to a history of premature death of family members. BMC Public Health.

[CIT0004] Arnberg F, Hultman C, Michel P, Lundin T (2013). Fifteen years after a ferry disaster: Clinical interviews and survivors’ self-assessment of their experience. European Journal of Psychotraumatology.

[CIT0005] Betancourt T. S (2015). The intergenerational effect of war. JAMA Psychiatry.

[CIT0006] Cloitre M, Garvert D, Brewin C, Bryant R, Maercker A (2013). Evidence for proposed ICD-11 PTSD and complex PTSD: A latent profile analysis. European Journal of Psychotraumatology.

[CIT0007] Cloitre M, Garvert D, Weiss B, Carlson E, Bryant R (2014). Distinguishing PTSD, Complex PTSD, and borderline personality disorder: A latent class analysis. European Journal of Psychotraumatology.

[CIT0008] Courtois C. A (2004). Complex trauma, complex reactions: Assessment and treatment. Psychotherapy: Theory, Research, Practice, Training.

[CIT0009] DeMause L (2002). The emotional life of nations.

[CIT0010] Duncan B, Miller S, Wampold B, Hubble M (2010). The heart and soul of change: Delivering what works in therapy.

[CIT0011] EU Treatises (1997). Amsterdam treaty. http://europa.eu/legislation_summaries/institutional_affairs/treaties/amsterdam_treaty/a16000_en.htm.

[CIT0012] Fischer G, Riedesser P (1999). Lehrbuch der Psychotraumatologie.

[CIT0013] Forsman A. K, Wahlbeck K, Aarø L. E, Alonso J, Barry M. M, Brunn M (2015). Research priorities for public mental health in Europe: Recommendations of the ROAMER project. European Journal of Public Health.

[CIT0014] Glassman M, Erdem G, Bartholomew M (2013). Action research and its history as an adult education movement for social change. Adult Education Quarterly.

[CIT0015] Grimm A, Hulse L, Preiss M, Schmidt S (2012). Post- and peritraumatic stress in disaster survivors: An explorative study about the influence of individual and event characteristics across different types of disasters. European Journal of Psychotraumatology.

[CIT0016] Herman J. L (1992). Complex PTSD: A syndrome in survivors of prolonged and repeated trauma. Journal of Traumatic Stress.

[CIT0017] Hicks D (2011). Dignity: The essential role it plays in resolving conflict.

[CIT0018] Jaspers K (1913). Allgemeine psychopathologie *[General psychopathology]*.

[CIT0019] Kluft R. P (2003). Antaeus and andragogy: Negotiating paradigm exhaustion and pursuing professional growth in clinical practice. American Journal of Clinical Hypnosis.

[CIT0020] Kuechenhoff J (2007). Dort wo ich berühre werde ich auch berührt [Where I touch there am I touched as well]. Forum der Psychoanalyse.

[CIT0021] Kuhn T. S (1962). The structure of scientific revolutions.

[CIT0022] Lacan J, Nobus D (1966). La Seminaire XIII, L'objet de la psychoanalyse (1965–1966). Unpublished seminar of 19 January 1966 (cited by B. Fink. (1998). The master signifier and the four discourses. Key concepts of Lacanian psychoanalysis.

[CIT0023] Levine P (1997). Waking the tiger.

[CIT0024] Lueger-Schuster B, Kantor V, Weindl D, Knefel M, Moy Y, Butollo A (2014). Institutional abuse of children in the Austrian Catholic Church: Types of abuse and impact on adult survivors’ current mental health. Child Abuse and Neglect.

[CIT0025] Middleton W, Stavropoulos P, Dorahy M. J, Krüger C, Fernandez-Lewis R, Martinez-Taboas A (2014a). The institutional abuse and societal silence: An emerging global problem. Australian and New Zealand Journal of Psychiatry.

[CIT0026] Middleton W, Stavropoulos P, Dorahy M. J, Krüger C, Fernandez-Lewis R, Martinez-Taboas A (2014b). The Australian Royal Commission into institutional responses to child sexual abuse. Australian and New Zealand Journal of Psychiatry.

[CIT0027] Nandi C, Crombach A, Bambonye M, Elbert T, Weierstall R (2015). Predictors of posttraumatic stress and appetitive aggression in active soldiers and former combatants. European Journal of Psychotraumatology.

[CIT0028] Newman R. D (1993). Transgressions of reading. Narrative engagement as exile and return.

[CIT0029] Olff M (2012). Advances in European Psychotraumatology. European Journal of Psychotraumatology.

[CIT0030] Olff M (2013). European Journal of Psychotraumatology (EJPT): Three years as an open access journal. European Journal of Psychotraumatology.

[CIT0031] Olff M (2014). Are we happy with the impact factor?. European Journal of Psychotraumatology.

[CIT0032] Olff M, Armour C, Brewin C, Cloitre M, Ford J. D, Herlihy J (2015). Trauma and PTSD: setting the research agenda. European Journal of Psychotraumatology.

[CIT0033] Olff M, Wall S (2014). Intimate partner violence and mental health—Remarks from two Chief Editors on a joint publishing venture. European Journal of Psychotraumatology.

[CIT0034] Orner R (2013). ESTSS at 20 years: “A phoenix gently rising from a lava flow of European trauma.”. European Journal of Psychotraumatology.

[CIT0035] Reifels L, Pietrantoni L, Prati G, Kim Y, Kilpatrick D, Dyb G (2013). Lessons learned about psychosocial responses to disaster and mass trauma: An international perspective. European Journal of Psychotraumatology.

[CIT0036] Sar V (2011). Developmental trauma, complex PTSD and the current proposal of DSM-5. European Journal of Psychotraumatology.

[CIT0037] Şar V, Krüger C, Martinez-Taboas A, Middleton W, Dorahy M (2013). Sociocognitive and posttraumatic models of dissociation are not opposed. Journal of Nervous and Mental Disease.

[CIT0038] Şar V, Middleton W, Dorahy M. J (2013). Individual and societal oppression: Global perspectives on dissociative disorders. Journal of Trauma and Dissociation.

[CIT0039] Sar V, Ozturk E (2005). What is trauma and dissociation?. Journal of Trauma Practice.

[CIT0040] Solinski S (2014). Betrayal in an age of terror. ISSTD Newsletter.

[CIT0041] Schnyder U (2013). Trauma is a global issue. European Journal of Psychotraumatology.

[CIT0042] Taleb N. N (2012). Antifragile: Things that gain from disorder.

[CIT0043] Thoresen S, Aakvaag H, Wentzel-Larsen T, Dyb G, Hjemdal O (2012). The day Norway cried: Proximity and distress in Norwegian citizens following the 22nd July 2011 terrorist attacks in Oslo and on Utøya Island. European Journal of Psychotraumatology.

[CIT0044] Van der Kolk B. A, Van der Kolk B. A, McFarlane A. C, Weisaeth L (1996). The complexity of adaptation to trauma: Self-regulation, stimulus discrimination, and characterological development. Traumatic stress.

[CIT0045] World Health Organization (1992). International classification of diseases.

